# Applicability of the theory of pain-sensorimotor interactions to orofacial pain and sensorimotor behavior, and implications for orofacial pain research and management

**DOI:** 10.22514/jofph.2025.066

**Published:** 2025-12-12

**Authors:** Barry J. Sessle, Greg M. Murray

**Affiliations:** ^1^Faculty of Dentistry and Temerty Faculty of Medicine Department of Physiology, and Centre for the Study of Pain, University of Toronto, Toronto, ON M5G 1G6, Canada; ^2^Discipline of Restorative and Reconstructive Dentistry, Sydney School of Dentistry, Faculty of Medicine and Health, The University of Sydney, 2006 Sydney, NSW, Australia

**Keywords:** Animals, Humans, Pain, Nociception, Motor activity, Neuronal plasticity, Psychosocial functioning, Genetics, Epigenomics, Electromyography

## Abstract

The orofacial sensorimotor system encompasses a variety of orofacial tissues as well as several pathways and circuits in the central nervous system (CNS) that participate in orofacial sensorimotor behaviors such as chewing, facial expression, speech, and swallowing. Acute or chronic pain can severely affect these behaviors, but the relationships between sensorimotor behaviors and pain, and the factors influencing the interactions and underlying mechanisms have remained unclear. Several theories proposed to account for the interactions and mechanisms have not provided a comprehensive picture of pain-sensorimotor interactions, nor fully acknowledged the diversity of biopsychosocial factors that may affect pain-related sensorimotor behaviors and the musculoskeletal tissues involved in the behaviors, and that may also contribute to the inter-individual and sex differences in pain-sensorimotor interactions. Such considerations prompted, last year, a review that has provided new perspectives of pain-sensorimotor interactions, and identified the wide range of biological, psychological, and sociocultural factors that may influence the interactions and underlying mechanisms. It also resulted in a novel theory being proposed, the Theory of Pain-Sensorimotor Interactions (TOPSMI), which has provided a more comprehensive mechanistic framework to consider the interactions between sensorimotor behaviors and pain, and their complexity. Since the new perspectives leading to TOPSMI were derived mainly from findings in the spinal sensorimotor system, the present article aims to determine the particular applicability of TOPSMI to orofacial pain-sensorimotor interactions. It reviews orofacial pain-related sensorimotor behaviors and the nociceptive pathways, CNS circuits and their plasticity, and musculoskeletal tissues involved in these behaviors, as well as the influences of psychosocial, genetic, epigenetic, and environmental factors bearing specifically on orofacial pain-sensorimotor interactions. The article concludes that the basic tenets of TOPSMI are applicable to orofacial pain-sensorimotor interactions and that this has implications for the clinical management of orofacial pain and future research in this field.

## 1. Introduction

The orofacial sensorimotor system encompasses a variety of tissues in the orofacial region as well as several pathways and circuits in the central nervous system (CNS) that participate in orofacial sensorimotor behaviors [1, 2, 3]. The orofacial tissues include skeletal muscles, bone, teeth, oral mucosa, facial skin, temporomandibular joints (TMJs) and neural elements. The central neural components of the system include a complex array of sensory and motor pathways, and neural circuits in the CNS that subserve the many sensory functions (*e.g.*, pain, touch, taste) and sensorimotor behaviors (*e.g.*, chewing, facial expression, speech, swallowing) that characterize the orofacial region. These sensorimotor behaviors can be influenced by pain, which is a complex multidimensional function defined as an “unpleasant sensory and emotional experience associated with, or resembling that associated with, actual or potential tissue damage” [4]. Pain may be acute or chronic, and both acute and chronic pain states are common in the mouth, face, and jaws [2, 5, 6, 7, 8]. Like pain states elsewhere in the body, orofacial pain can encompass sensory-discriminative, motivational-affective, and cognitive-evaluative dimensions [1, 6]. These three dimensions are particularly evident in states of chronic pain (defined as pain present for at least 3 months [6, 7, 9]), and their complex inter-relationships can make it challenging for clinicians to provide timely diagnosis and effective management for the individual experiencing chronic pain. Adding to the challenge is that pain, particularly chronic pain, can frequently be accompanied by one or more comorbidities such as psychosocial problems (*e.g.*, stress, anxiety, depression), disturbed sleep, and pain manifested elsewhere in the body, as well as by disruptions in skeletal muscle functions involved in sensorimotor behaviors such as those related to walking or eating.

The interactions and relationships between pain and sensorimotor behaviors and the factors influencing them and the underlying mechanisms have been a particular focus of studies and discussion for several decades. A number of theories have been put forward over this period to account for the interactions and their expression in skeletal muscle functions, and to provide a rationale for clinical procedures advocated for the treatment of pain states. Of the several theories developed, the pain-spasm-pain vicious cycle (commonly termed the Vicious Cycle Theory, VCT) and the Pain Adaptation Model (PAM) have attracted the most attention [8, 10, 11, 12]. These two theories propose that pain results in reflex changes in electromyographic (EMG) activity that are stereotypical in the sense that the same hypothesized changes in EMG activity (*i.e.*, increases or decreases) occur throughout all functional groupings of muscles in a sensorimotor system utilizing these muscles. While the simplicity of the VCT and PAM has made them readily interpretable by clinicians and patients to justify particular modes of treatment in the management of pain, some modes of treatment have provided outcomes that are sometimes no better than the placebo or conservative management approaches [10, 13]. Furthermore, a number of reports have detailed significant limitations in the VCT and PAM and many clinical or experimental pain findings are inconsistent with both theories [8, 11, 12]. In addition, both theories are incongruous by inferring that a nociceptive reflex associated with changes in muscle activity can be evoked by pain *per se*. This is because the multi-dimensional experience of pain primarily involves circuitry at higher (*i.e.*, cerebral cortical) levels of the CNS, whereas nociceptive reflexes have their central circuitry residing at segmental brainstem or spinal cord levels of the CNS. A nociceptive reflex can be a feature of the pain experience, but a nociceptive reflex cannot be evoked by pain itself because a nociceptive reflex is the segmental motor response to a noxious stimulus that evokes nociceptive afferent inputs that access the CNS.

Several other theories have been proposed in recent decades to help explain some of the observations from clinical and experimental pain investigations of pain-sensorimotor interactions and to account for the possible role of other factors (*e.g.*, psychological) in these interactions. These theories include the Fear-Avoidance Model of Pain, the Avoidance-Endurance Model, the Integrated Pain Adaptation Model, and the New or Contemporary Theory for the Motor Adaptation to Pain [8, 11, 12]. While some of these theories have led to improvements in patient management, none of them, nor the earlier VCT or PAM, has provided a full picture of pain-sensorimotor interactions that recognizes the broad range of biological, psychological, and sociocultural factors and mechanisms that may influence pain-sensorimotor interactions. In particular, they do not clearly acknowledge the importance of genetic, epigenetic, and allied environmental factors as well as many psychosocial influences that may affect the experience of pain, including pain-related sensorimotor behaviors and the musculoskeletal tissues involved in the behaviors, and that also may be involved in the inter-individual and sex differences in the pain experience. Such considerations prompted last year a review [14] that has provided new perspectives of pain-sensorimotor interactions that have been formulated in accordance with the biopsychosocial model of pain [15, 16] depicted in Fig. 1. The review identified the diverse range of biological, psychological, and sociocultural factors that may influence the interactions and the underlying mechanisms. It also resulted in a novel theory being proposed, the Theory of Pain-Sensorimotor Interactions (TOPSMI), which has provided a more comprehensive and mechanistic framework to clarify the interactions between pain and sensorimotor behaviors [14]. The TOPSMI proposes that “*pain is associated with plastic changes in the central nervous system (CNS) that lead to an activation pattern of motor units that contributes to the individual’s adaptive sensorimotor behavior. This activation pattern takes account of the biological, psychological, and social influences on the musculoskeletal tissues involved in sensorimotor behavior and on the plastic changes and the experience of pain in that individual. The pattern is normally optimized in terms of biomechanical advantage and metabolic cost related to the features of the individual’s musculoskeletal tissues and aims to minimize pain and any associated sensorimotor changes, and thereby maintain homeostasis. However, adverse biopsychosocial factors and their interactions may result in plastic CNS changes leading to less optimal, even maladaptive, sensorimotor changes producing motor unit activation patterns associated with the development of further pain*” [14].

**Fig. 1.** Examples of the biological, psychological, and sociocultural components that contribute to the biopsychosocial model of pain. These components, and interactions among the components, can influence pain features and result in a mosaic of features that differ between individuals.

The new perspectives leading to TOPSMI were based principally on findings from studies in animal models and humans related to pain and pain-sensorimotor interactions in the spinal nociceptive and sensorimotor systems (hereafter termed “the spinal system”). They outlined the features and factors bearing on the interactions, namely nociceptive pathways, CNS circuits and their plasticity, musculoskeletal tissues, and the influences of psychosocial, genetic, epigenetic, and environmental factors. The present article builds upon these findings with the aim to determine the particular applicability of TOPSMI to orofacial pain-sensorimotor interactions. It reviews the features and factors derived from investigations in animals and humans which relate specifically to orofacial pain and associated sensorimotor interactions involving orofacial skeletal muscles and, in a novel analysis, considers them in turn for their applicability to the basic tenets of TOPSMI. Since most pain-sensorimotor interaction studies have centered on the spinal system, each of the following sections dealing in turn with findings from studies in animals and in humans includes first a brief overview of relevant findings in the spinal system, followed by a more extensive detailed review of findings from studies of orofacial pain-sensorimotor interactions. Some attention is also given to the clinical implications of TOPSMI for the diagnosis and management of orofacial pain-related sensorimotor disorders. In addition, some consideration is given to possible future research avenues to provide further insights and also test the applicability of TOPSMI to explaining orofacial pain-sensorimotor interactions. Pain-related sensorimotor effects associated with the autonomic innervation of tissues (glands; cardiac or smooth muscle) are not considered for this article.

## 2. Orofacial pain and related sensorimotor behaviors

### 2.1 Animal models

In the spinal system, studies in animals of pain and pain-sensorimotor interactions have revealed a diverse variety of pain-related behaviors, many of which mimic the features of allodynia, hyperalgesia, and extraterritorial spread of sensitivity in several acute and chronic pain states in humans [14, 17, 18, 19, 20, 21]. The animal models of acute pain commonly have employed a brief delivery of noxious stimuli to tissues innervated by nociceptive primary afferent nerve fibers that may produce a range of reflex sensorimotor behaviors (*e.g.*, limb withdrawal, paw lift, tail flick, flinching). These reflex behaviors depend on relatively simple, segmentally based CNS circuits and have been widely used to explore nociceptive mechanisms, neuroplasticity, glioplasticity, and modifications to gene expression in peripheral tissues as well as the CNS. The chronic pain models in animals commonly demonstrate evoked or spontaneous pain-like behaviors as well as behaviors reflecting motivational or affective aspects of pain (*e.g.*, stress, anxiety), survival behaviors (*e.g.*, avoidance), natural or elective behaviors (*e.g.*, social interaction, nest building, wheel running), and/or other sensorimotor behavioral responses to noxious stimuli (*e.g.*, vocalizations, guarding, licking, writhing). Furthermore, inter-individual variability and age and sex differences in pain-related sensorimotor behaviors have been well-documented in these acute and chronic pain models in the spinal system, with a wide range of contributing influences that include biological, psychological, and sociocultural factors that may also affect musculoskeletal tissues themselves, as detailed in sections 4 and 5.

In the case of sensorimotor behaviors related to acute orofacial pain, animal studies have particularly focused on orofacial nociceptive or inflammatory pain models using noxious mechanical (*e.g.*, pinch, heavy orthodontic forces), noxious thermal (*e.g.*, radiant heat) or algesic chemical (*e.g.*, hypertonic saline, capsaicin, mustard oil) stimuli delivered to superficial tissues (*e.g.*, oral mucosa, cornea, facial skin) or deep tissues (*e.g.*, jaw muscle, tooth pulp, temporomandibular joint (TMJ), meninges) supplied by the trigeminal nociceptive afferents [1, 2, 6, 21, 22, 23, 24, 25]. Activation of these afferents typically evokes reflex sensorimotor behaviors, for instance the jaw-opening reflex, that manifests as reflex jaw opening reflecting increased EMG activity of muscles involved in jaw-opening (*e.g.*, anterior digastric) and decreased EMG activity of muscles involved in jaw-closing (*e.g.*, masseter), and other reflex changes which may occur in facial, tongue, and neck muscles. However, orofacial noxious stimulation in animal pain models may sometimes lead to robust increases in EMG activity of both jaw-opening and jaw-closing muscles via N-methyl-D-aspartate (NMDA) receptor-dependent circuits as well as recruitment of other circuits and processes (*e.g.*, central γ-amino-butyric acid (GABA) and opioid mechanisms) that may modulate the neural activity driving the EMG activity [2, 22, 26]. Furthermore, these various reflex responses to orofacial noxious stimulation are primarily based on brainstem circuits and have been used to identify some of the pathways and interneuronal circuits through which the higher centers of the brain can modulate orofacial sensorimotor behaviors or to document the crucial role played by components of the trigeminal brainstem sensory nuclear complex (VBSNC) and adjacent brainstem sites in these circuits and modulatory influences (see section 3.1).

In terms of chronic orofacial pain, several animal models have been formulated in relation to inflammatory pain-related behaviors induced by sustained mechanical noxious stimulation (*e.g.*, placement of an occlusal interference, ligation of masseter muscle tendon, maintained jaw opening) or the delivery to orofacial tissues of inflammogens or chemical irritants having a prolonged action (*e.g.*, formalin, carrageenan, complete Freund’s adjuvant (CFA), cytokines) [6, 22, 23, 24, 25, 26, 27, 28, 29]. These models have been quite informative in providing insights into animal behaviors and peripheral and central mechanisms related to chronic pain. However, a limitation of many is that the injection or tissue injury may be restricted to a confined tissue area whereas in several chronic pain conditions in humans, the pain expressed is more extensive. Nonetheless, these chronic pain models have revealed that, compared with the models of acute pain, a more complex CNS circuitry is engaged that underlies an array of spontaneous or evoked behaviors [6, 22, 23, 24, 25]. These behaviors reflect features such as nocifensive withdrawal responses to mechanical, thermal or chemical stimuli that may be indicative of allodynia or hyperalgesia which may last many hours or several days or weeks; changes may also occur in grooming, exploratory, licking and guarding behaviors, facial grimacing, biting and gnawing, and disruptions may occur in chewing, drinking or other sensorimotor behaviors, and also operant motivational and affective pain-related behaviors. Many analogous behaviors occur in animal models of orofacial neuropathic pain typically produced by mechanical injuries to nerves (*e.g.*, placement of loose ligatures around a nerve, chronic constriction injury model or sectioning of part or all of a peripheral nerve) [6, 27, 30]. Neuropathic pain models also have included methods inducing viral infections (*e.g.*, herpes virus introduced into the trigeminal ganglion to model trigeminal post-herpetic neuralgia) or compression of the trigeminal sensory root or trigeminal ganglion or disturbance of trigeminal CNS pathways (to model some trigeminal neuropathic pain states, *e.g.*, trigeminal neuralgia) [6, 27, 30]. Some orofacial pain models have also been developed that may involve both inflammatory and neuropathic components, as in cancer pain [23], but more research is needed to clarify the mechanisms underlying the pain and related behaviors associated with orofacial cancers.

Similar to observations in the animal pain models employed in the spinal system, inter-individual variability and age and sex differences in sensorimotor behaviors related to pain may occur in the animal models of acute or chronic orofacial pain [6, 20, 21, 22, 29, 31, 32, 33, 34, 35]. For example, in comparison with male rats, female rats exhibit greater nociceptive afferent excitability and jaw EMG activity following glutamate or formalin application to the TMJ or jaw muscle, and considerable variability within a study may occur in recorded variables between animals receiving the same noxious stimulus. Some of the differences and variability can be attributed to factors that may not have been specifically studied in these experiments, such as genetic, psychosocial, and environmental factors which are discussed further below (sections 4 and 5). Other contributory factors could be changes in musculoskeletal tissues. For example, these tissues may show age-related changes as well as alterations induced by environmental factors such as tissue injury, tooth loss, and drugs [20, 21, 36, 37]. In addition, functional and dysfunctional sensorimotor behaviors involving jaw, lip or tongue can affect the dentition, TMJ, and other orofacial musculoskeletal tissues in animals, and changes in pain-related sensorimotor behaviors can lead to morphological and biochemical changes in muscles and joints [14, 24, 25].

### 2.2 Human studies

While valuable in revealing circuits and mechanisms involved in pain, some of the findings from animal pain models may not be readily translatable to human acute and chronic pain states or to effective clinical therapies for pain relief [17, 19]. This underscores the need for complementary experimental studies, where feasible in humans, to determine the relevance of findings from animal pain models to human pain states [17, 18]. Human spinal system studies focusing on pain and related sensorimotor behaviors have principally involved investigations delivering noxious stimuli in experimental paradigms of acute pain in healthy individuals who are free of pain or studies of individuals experiencing acute pain (*e.g.*, arthritis, myositis, acute gastrointestinal tract inflammation) or chronic pain (*e.g.*, low back pain, fibromyalgia, neuropathic pain) [12, 14, 15, 18, 38]. The acute pain experiments typically have applied brief noxious stimuli to tissues supplied by primary afferent nerve fibers that can evoke reflex kinematic sensorimotor behaviors (*e.g.*, limb withdrawal) or changes in EMG activity. The chronic pain experiments have commonly recorded changes in behavioral features (*e.g.*, limb or spine kinematics or EMG activity). Both acute and chronic pain studies have included corticospinal excitability and brain-imaging investigations of the cerebral cortical and other areas of the CNS that have been implicated in sensorimotor behaviors related to pain (see section 3.2). These studies have provided not only insights into the sensorimotor behaviors associated with acute and chronic pain but also evidence for the behaviors influencing and being influenced by musculoskeletal tissues. There is also evidence for a large variation between individuals as well as age and sex differences in pain features (*e.g.*, pain threshold, intensity, tolerance) and in some motor outcome measures including task kinematics and reorganization or redistribution of EMG activity within and between muscles in acute or chronic pain states [14, 15, 38].

Investigations of acute orofacial pain and related sensorimotor behaviors in humans have demonstrated many features analogous to those identified for pain and related sensorimotor behaviors in studies of the spinal system [1, 2, 5, 8, 10, 15, 20, 21, 39, 40, 41]. Some of these investigations have documented pain characteristics and behaviors of patients with acute pain states (*e.g.*, post-extraction pain), others have used noxious stimuli in experimental paradigms of acute pain in pain-free healthy individuals; these stimuli have included an algesic chemical (*e.g.*, capsaicin, hypertonic saline, glutamate, buffered acidic saline) applied topically or by injection to skin, muscle or TMJ, and electrical or intense thermal or mechanical stimulation of skin, muscle, TMJ or teeth. These various studies have employed a variety of recording methodologies, including EMG recordings during excitatory or inhibitory reflexes evoked in tongue (*e.g.*, genioglossus), facial (*e.g.*, orbicularis oculi), jaw-closing (*e.g.*, masseter, temporalis) or jaw-opening (*e.g.*, anterior digastric) muscles, EMG recordings during postural or rest position of the lower jaw, and recordings of face, tongue, or jaw movements, forces, and/or EMG activity during motor task performance (*e.g.*, jaw closing and opening, biting, lip or tongue protrusion, or rhythmical movements such as chewing). Several studies have delivered orofacial noxious stimuli during recordings of evoked motor activity in relation to corticobulbar excitability, organizational or neuronal activity properties of the cortical face primary motor area (face M1) or other CNS areas (see section 3.2). While these studies have been important in advancing understanding, there remains the need for improved methodologies for recording of EMG activity, kinematics and dynamics of face, and jaw and tongue movements to provide more comprehensive and fine-grained data sets that will facilitate a more detailed characterization of orofacial pain-related sensorimotor changes. Such approaches will undoubtedly benefit from the use of artificial intelligence (AI)-assisted analyses of the recorded data [42, 43]. Other studies using subjective measures of jaw motor behaviors (*e.g.*, via questionnaires) to investigate associations with pain will not be considered in detail since this present article focuses on objective measures of motor behaviors.

Studies of acute orofacial pain and tongue or face motor activity [1, 21, 40, 41] have included those documenting excitatory and/or inhibitory reflexes in tongue muscles (*e.g.*, genioglossus) and facial muscles (*e.g.*, orbicularis oculi) induced by noxious orofacial stimuli. Other studies have shown that moderate pain evoked by topical capsaicin application to the tongue is associated with a reduction in motor performance of a tongue-protrusion task, and, consistent with findings of reduced face M1 excitability with noxious orofacial stimulation in animals, a disruption of the face M1 cortical neuroplasticity underlying the task learning [5, 21] (see section 3.2).

In terms of jaw motor activity in relation to acute orofacial pain, numerous studies have shown that brief experimental noxious stimulation of orofacial tissues typically evokes a jaw-opening reflex involving excitation of jaw-opening muscles and inhibition of jaw-closing muscles [1, 2, 8, 39]. There is no clear consensus as to whether more prolonged noxious orofacial stimulation is associated with an increase, a decrease, or no change in resting (postural) jaw muscle EMG activity in comparison with pain-free control [8, 10, 39]. However, it is clear that noxious stimulation can interrupt or alter ongoing orofacial motor activities during chewing and speech [2, 8, 10, 39], and that algesic chemical intramuscular injection into a jaw-closing muscle (masseter or temporalis) or the splenius muscle can facilitate the jaw-closing reflex recorded as masseter or temporalis EMG activity and inhibit the exteroceptive suppression (evoked by noxious facial skin stimulation) of masseter and temporalis EMG activity during standardized jaw clenching [44, 45, 46, 47]. Although the facilitation of the jaw-closing reflex noted in some of these human studies during experimental acute noxious stimulation is consistent with the robust excitation of jaw closing muscles with noxious orofacial stimulation in animal experiments (see section 2.1), it is not possible to draw a similar comparison between these human and animal studies with regards to the antagonist (jaw-opening) muscles as these particular human studies, in contrast to the animal studies, did not make simultaneous recordings from the jaw-opening muscles, *e.g.*, the anterior digastric or lateral pterygoid muscles. Other studies of the effects of experimental acute orofacial noxious stimulation on jaw movements, force generation and/or EMG activity during jaw motor tasks have reported effects (*e.g.*, an increase, a decrease or no significant change) that vary between studies, tasks, subjects, and muscles [8, 39, 48, 49]. Nonetheless, consistent with the related spinal literature, experimental acute noxious stimulation may result in a reorganization or a redistribution of motor unit activity as evidenced by, for example, recruitments and de-recruitments of single motor units within the same jaw muscle, irrespective of whether that particular muscle has been subjected to the noxious stimulation or not [50, 51].

In the case of pain-related sensorimotor behaviors in chronic orofacial pain, several studies have documented pain characteristics and behaviors of patients with a diagnosed orofacial chronic pain state (*e.g.*, temporomandibular disorders (TMD), persistent idiopathic facial pain, persistent dentoalveolar pain, burning mouth syndrome (BMS), migraine) [5, 6, 8, 15, 39, 40, 52, 53]. Like the studies of acute orofacial pain and sensorimotor behavior, these investigations have typically involved recordings of movement kinematics or EMG activity during motor tasks or evoked orofacial reflexes, and some have investigated experimentally induced pain in asymptomatic subjects (*e.g.*, through single or repeated injections of nerve growth factor (NGF) into a jaw muscle or repetitive jaw muscle contractions evoking delayed onset muscle soreness) that may last longer than that for the experimental acute orofacial pain models and thereby may more closely reflect some features of chronic pain. Other orofacial chronic pain investigations have also imaged areas of the CNS implicated in pain-related sensorimotor behaviors or have studied corticobulbar excitability (see section 3.2), and some recent studies have adopted “AI” methodologies to assist in the analysis of CNS imaging findings or clinical history in order to facilitate mechanistic understanding as well as the diagnosis and assessment of orofacial pain states [42, 43].

Changes in face or tongue motor behaviors in chronic orofacial pain include attenuation of components of the nociceptive blink reflex in BMS, persistent dentoalveolar pain, persistent idiopathic facial pain, and migraine [40, 53]. It has been suggested that such attenuation may reflect reduced efficiency of the descending pain-modulatory pathways in exerting inhibition within CNS nociceptive circuits [53]. Pain-related changes in jaw motor behaviors are less clear. For example, it has been reported that resting (postural) EMG activity of masseter and temporalis muscles is significantly higher, with jaw-closing reflex EMG amplitude being significantly lower in patients with a trigeminal neuropathic pain state [54], but the possible contamination of the jaw muscle EMG signals by facial muscle activation is a limitation of these findings [55]. Investigations of jaw motor behaviors during other chronic orofacial pain states (mostly TMD) have produced conflicting findings, with some reporting no significant change in jaw muscle EMG activity in postural jaw position and in jaw movements, force generation and/or EMG activity during a variety of jaw motor tasks, and others reporting a significant increase or decrease [8, 10, 39, 52]. It has been suggested that methodological differences between studies or contaminations of the EMG recordings from activation of facial muscles may have contributed to the lack of consensus between studies [10, 52, 55]. The possible role of other factors (*e.g.*, genetic, psychosocial) not specifically investigated in most of these studies may also have contributed to the discrepancies in the reported EMG activity patterns related to orofacial pain.

Like chronic pain states in the spinal system, a number of orofacial chronic pain states (*e.g.*, migraine, some neuropathic pain states, TMD) have a female predominance, and there is variation between individuals and males and females in some pain features (*e.g.*, pain location, pain intensity) [6, 15, 31, 32, 33, 34, 35]. Sex or inter-individual differences have been reported in human studies for some masticatory cycle parameters [56] and single motor unit functional properties [57]. However, while some inter-individual variability and sex differences in sensorimotor behaviors related to pain have been documented in both acute and chronic orofacial animal pain models as noted above, there is limited and unclear information if these differences occur in humans. For example, there is inconsistency between studies as to whether sex differences exist in nociceptive blink and jaw motor behaviors [40, 47, 48, 53, 58]. Nonetheless, there is evidence of significantly greater variability in some jaw movement kinematic features between individuals with a past history of neuropathic pain *vs*. matched controls [59], and the occurrence of inter-individual differences is also suggested by variability in experimental acute pain-related behavioral effects [49], and between individuals with the same diagnosed chronic pain state in their adapted sensorimotor behaviors [60]. Another possible contributing factor could be an age difference of the subjects engaged in the different studies since significant effects of ageing have been reported on orofacial sensorimotor functions, including age-related alterations of masticatory, swallowing, speech motor activity, bite force, and EMG activity associated with jaw-closing and blink reflexes [21, 58]. The possible presence of age as well as inter-individual and sex differences in orofacial pain-sensorimotor interactions is an area warranting further research in view of its relevance to clinical diagnosis and management of pain-related sensorimotor behaviors.

It is also noteworthy from studies in humans as well as animal models (see above) that functional or dysfunctional orofacial sensorimotor behaviors can influence orofacial musculoskeletal tissues. For example, facial (lip) and tongue pressures contribute to determining dental arch form, and bruxism may affect dental occlusal and mandibular morphology [1, 41, 61, 62]. Moreover, these tissues may be affected by orofacial pain-related sensorimotor activity, for example where maladaptive alterations in jaw posture or movements in the presence of pain in some individuals might produce changes in dental and other orofacial musculoskeletal tissues [13, 14, 63]. These effects could contribute to some of the inter-individual and sex differences noted above.

In summary, it is clear from the observations in animal models and humans that both acute and chronic orofacial pain states are associated with a wide range of sensorimotor effects in face, tongue, and jaw motor activity, and could involve structural and metabolic changes in orofacial musculoskeletal tissues as well as in related CNS circuits. There may however be variability between individuals and age-related, and possibly sex, differences in these sensorimotor effects and other pain features. Several factors may contribute to these differences, and are considered further in sections 4 and 5. The range of possible pain features and motor patterns engaged in sensorimotor behaviors in the presence of noxious orofacial stimulation or pain is consistent with the TOPSMI by way of its enunciation of the many motor patterns and factors at play in the interactions between pain and sensorimotor behaviors. Underpinning these behavioral effects are the nociceptive pathways and their circuits within the CNS as well as the crucial role played in these pathways and circuits by neuroplastic and glioplastic changes, some of which may be maladaptive and disrupt the normal functions of these CNS pathways and circuits; these aspects will now be reviewed.

## 3. Orofacial nociceptive pathways, sensorimotor circuits, and their plasticity

### 3.1 Animal models

In the spinal system, a variety of techniques has been developed and applied in animal models of acute or chronic pain to investigate neural pathways, CNS circuits, and underlying mechanisms (*e.g.*, plasticity) contributing to the pain-related sensorimotor behaviors described above (section 2.1). These techniques, which also have been applied for comparable orofacial pain investigations (see below), include electrophysiological, neuroanatomical, immunohistochemical, and genetic approaches applied *in vivo* and/or *in vitro* [14, 17, 18, 64, 65, 66]. Many of these studies have revealed that noxious stimuli producing injury or inflammation of peripheral tissues can activate the nociceptive endings (nociceptors) of some small-diameter (A-δ and C-fiber) spinal primary afferent nerve fibers innervating these tissues. The activation commonly occurs indirectly, *i.e.*, through chemical mediators (*e.g.*, adenosine triphosphate (ATP), glutamate) released from the damaged or inflamed tissues and the nociceptors themselves, and these chemical mediators act upon specific ion channels or membrane receptors on the nociceptors to induce increased nociceptor excitability and the elicitation of nociceptive signals in the form of action potentials. Some noxious stimuli (*e.g.*, heat) can instead act directly on specific ion channels on the nociceptor. It has been shown that the nociceptive signals are conveyed from the activated nociceptors along the spinal nociceptive afferents via their cell bodies (in the dorsal root ganglia (DRG)) into the CNS where the signals are principally processed in and transmitted through the dorsal horn of the spinal cord to many spinal and supra-spinal regions, including the ventral horn, brainstem reticular formation, parabrachial nucleus, cerebellum, rostral ventromedial medulla (RVM), periaqueductal gray matter (PAG), and thalamus. From the thalamus, the signals are relayed to and processed in areas of the cerebral cortex. These CNS areas contribute to the various dimensions of pain and to descending pain-modulatory pathways that can modulate nociceptive transmission and thereby have an effect on sensorimotor behaviors. Furthermore, inter-individual and sex differences have been well-described in some of these nociceptive circuits and processes.

Extensive glioplasticity, neuroplasticity, and neuroimmune interactions may accompany tissue injury and inflammation [14, 18, 64, 65, 66]. The plasticity and interactions occur in nociceptors (and their DRG cell bodies) where they can manifest as an increased nociceptor excitability (*i.e.*, peripheral sensitization) typically produced by some of the chemical mediators released from the injured or inflamed tissues, afferent endings or blood vessels (*e.g.*, ATP, glutamate, cytokines, 5-hydroxytryptamine (5-HT, serotonin), histamine, substance P) and acting on their respective ion channels and membrane receptors on the nociceptors; some released mediators instead reduce excitability (*e.g.*, enkephalins, cannabinoids, GABA). The nociceptive afferent inputs into the CNS generated by tissue injury or inflammation can also induce neuroplastic changes in the CNS nociceptive pathways such as the spinal cord dorsal horn, where they are reflected in an increased excitability of nociceptive neuron as so-called central sensitization. The neuroplastic changes are accompanied by and indeed dependent on morphological and functional changes occurring in local glial cells (*e.g.*, astrocytes, microglia) and neuroimmune interactions. Along with peripheral sensitization, central sensitization is considered to account for the allodynia, hyperalgesia, and pain spread as well as pain-related behaviors that are commonly seen in acute or chronic pain states (*e.g.*, low back pain, arthritis, toothache). Inter-individual and sex and age differences occur in some of these processes, and there is evidence that some of the CNS plasticity changes may represent a maladaptive plasticity which is defined here as plasticity that disrupts or compromises the normal function of the CNS regions affected by the plastic changes [20, 64, 66].

In the orofacial nociceptive and sensorimotor systems, the trigeminal nerve is the major nerve supplying orofacial tissues, with its primary afferent fibers ending as sensory receptors (*e.g.*, low-threshold mechanoreceptors, thermoreceptors, proprioceptors, nociceptors) in these tissues, and its efferent fibers terminating in many of the jaw muscles [1, 2, 3] (Fig. 2, Ref. [21]). Other cranial nerves (*e.g.*, VII, IX, X, XII) provide afferent innervation to other sensory receptors in these tissues (*e.g.*, lingual taste buds, pharyngeal mechanoreceptors and chemoreceptors) or efferent innervation of other muscles (*e.g.*, facial, lingual, pharyngeal) (Fig. 2). In the case of acute orofacial pain, trigeminal nociceptors, like their counterparts in the spinal system, can be activated by a variety of noxious stimuli that can directly or indirectly activate them or modulate their excitability, producing peripheral sensitization for example; there also are sex differences in some of these features, as noted below. The A-δ and C-fiber primary afferents associated with the nociceptors convey nociceptive signals generated by the nociceptors through the trigeminal ganglion (where their cell bodies are located) to the rostral parts (*e.g.*, subnucleus oralis) of the VBSNC and especially to its most caudal component, subnucleus caudalis (Vc), which is often termed the medullary dorsal horn in view of its several structural and functional similarities with the dorsal horn of the spinal cord; some signals may also be conveyed to adjacent regions (*e.g.*, C1-2 spinal dorsal horn, transitional zone between Vc and VBSNC subnucleus interpolaris, reticular formation) [1, 2, 6, 22, 23, 24, 26, 27, 28, 30, 67, 68, 69]. Trigeminal afferents carrying signals from most orofacial low-threshold mechanoreceptors, thermoreceptors, and proprioceptors project to the rostral and caudal components of the VBSNC, whereas taste-related signals carried by cranial nerve VII, IX and X afferents are mainly processed in the solitary tract nucleus. These various signals are relayed to motoneurons located within brainstem motor nuclei (*e.g.*, trigeminal, facial, and hypoglossal motor nuclei) and/or to neurons in other regions of the brainstem (*e.g.*, parabrachial nucleus, RVM, and reticular formation, parts of which contribute to the circuits serving as the central pattern generators for mastication and swallowing) (Fig. 2). There is evidence that neurons within specific parts of the VBSNC (*e.g.*, Vc; VBSNC subnucleus interpolaris, the transitional zone between Vc and subnucleus interpolaris) as well as in regions adjacent to VBSNC and motor nuclei receive these signals and serve as interneurons in orofacial reflexes and other sensorimotor behaviors [2, 6, 21, 26, 27, 30, 69]. In addition, these signals are also relayed to higher subcortical and cortical areas of the CNS (*e.g.*, somatosensory thalamus, hypothalamus, amygdala, cerebral cortex) for further processing [2, 6, 26, 27, 67] (Fig. 2).

**Fig. 2.** Schematic diagram of some of the main CNS neural circuits involved in orofacial sensorimotor integration and the modulatory influences affecting them. This schematic diagram illustrates some of the important afferent pathways from the orofacial tissues to the CNS and the efferent pathways from the CNS to face, jaw and tongue muscles. Also illustrated are some of the major CNS areas and pathways involved in sensorimotor processing, integration, modulation and control. Motor V, VII, and XII: motor nuclei of the trigeminal, facial, and hypoglossal cranial nerves, respectively; Sensory V: trigeminal brainstem sensory nuclear complex; STN: solitary tract nucleus. From “Jaw sensorimotor control in healthy adults and effects of ageing”, L. Avivi-Arber and B.J. Sessle, Journal of Oral Rehabilitation 45:1, Copyright© (2018), Wiley, John Wiley & Sons Ltd [21].

Different areas of the cerebral cortex are involved in the different aspects of orofacial pain, including sensorimotor behaviors [5, 6, 26, 67] (Fig. 2). For example, regions representing the face in the primary somatosensory area (S1) and secondary somatosensory area (S2) are particularly involved in the sensory-discriminative aspect of orofacial pain and also in sensorimotor behaviors related to orofacial pain. Also, the cortical face primary motor area (face M1) is involved in voluntary orofacial sensorimotor control, including motor responses to noxious stimuli; the face S1 and face M1 are often collectively termed the face sensorimotor cortex [5]. Other cortical areas, such as prefrontal cortex (PFC), anterior cingulate cortex (ACC), and insula, are more involved in the cognitive-evaluative, motivational-affective and/or emotional aspects of pain. Most of these cortical areas communicate with each other and with subcortical regions such as the hypothalamus, amygdala, reticular formation, RVM, and PAG which are components of the descending pain-modulatory pathways [6, 26, 67]. Through their release of a variety of neurochemicals (*e.g.*, enkephalins, 5-HT, noradrenaline), these different components exert facilitatory or inhibitory influences on nociceptive transmission including that in the VBSNC. These pain-modulatory influences represent CNS processes by which factors such as stress, anxiety, pain catastrophizing, pain expectations, depression, and some environmental factors could enhance pain and related sensorimotor behaviors, and by which therapeutic procedures such as cognitive behavioral therapy, acupuncture, motor cortex or deep brain stimulation, and many analgesic drugs could reduce the pain experience and also modify pain-related behaviors [14, 26, 67]. The descending pain-modulatory pathways have also been implicated in the phenomenon, seen in animals, of diffuse noxious inhibitory controls (DNIC) which is equivalent in humans to conditioned pain modulation (CPM), whereby an acute noxious stimulus delivered to a body site (*e.g.*, limb) suppresses nociceptive transmission (*e.g.*, in Vc and C1-2), pain and pain-related behaviors induced by noxious stimulation of another spatially remote site (*e.g.*, face) [26, 67, 70].

Acute orofacial noxious stimuli may result in the activation of two main types of nociceptive neurons in the CNS (*i.e.*, nociceptive-specific; wide dynamic range), and the excitability of these neurons can be increased through a “wind-up” of nociceptive neuronal responsiveness to repeated acute noxious stimuli [2, 6, 27, 68, 71]. A more prolonged increase in excitability expressed as central sensitization of these nociceptive neurons may occur as a result of neuroplastic and glioplastic changes induced by the nociceptive afferent inputs evoked by noxious stimuli. The neuroplastic changes have been especially well-documented in Vc/C1-2 nociceptive neurons and shown to be dependent on Vc/C1-2 glioplasticity and to involve neuroimmune interactions [2, 6, 26, 27, 68, 71]. The central sensitization stemming from these plastic processes is associated with pain-like behaviors and alterations in masticatory behaviors, and is crucial not only for the development but also the maintenance of chronic pain [2, 6, 26, 27, 68, 71]. Orofacial noxious stimulation can also induce plastic changes and increased excitability at other levels of the trigeminal nociceptive pathways (*e.g.*, subnucleus oralis, thalamus, face sensorimotor cortex) [5, 6].

Studies using animals to model chronic orofacial pain also have documented neuroplastic changes in Vc/C1-2 nociceptive neurons and some other components of trigeminal nociceptive pathways as well as in CNS areas involved in sensorimotor, cognitive-evaluative, and motivational-affective functions and pain modulation [2, 6, 27, 67, 72]. Central sensitization may result from the Vc/C1-2 neuroplastic changes; it is accompanied by chronic pain-like behaviors and has several features similar to those in acute pain models, including the involvement of neuroimmune interactions and dependency on glial cell plasticity [6, 26, 27, 29, 67, 68, 69, 71]. The plastic changes occurring in the other CNS areas may include disruption of the normal functioning of the descending pain-modulatory pathways and thereby reflect a maladaptive plasticity that plays a crucial role in the development and maintenance of chronic pain states. This is exemplified by findings that prolonged masseter inflammation produces persistent orofacial nociceptive sensorimotor behaviors associated with neuroplasticity and glioplasticity in the caudal VBSNC and alterations in the RVM descending modulatory influences on VBSNC nociceptive processes [29, 68, 69, 71]. Furthermore, the placement of a dental occlusal interference can induce neuroplastic changes reflected in central sensitization in caudal VBSNC as well as RVM plastic changes in association with long-lasting orofacial nociceptive sensorimotor behaviors, which can persist unless the occlusal interference is removed within a few days of its placement [28]. It is noteworthy that neuroplastic and glioplastic changes in the CNS can occur in states other than those associated with pain. For example, CNS plasticity is a fundamental process occurring during ageing, and is also necessary for functioning of CNS areas involved in sensorimotor task learning and in memory, and orofacial manipulations such as trimming or extraction of teeth or facial whiskers, and orthodontic tooth movement can induce plastic changes in several of the CNS areas noted above, including face sensorimotor cortex [5, 20, 21, 72, 73].

It is also notable that age and inter-individual and sex differences may exist in the orofacial nociceptive and sensorimotor systems [6, 20, 22, 31, 32, 33, 34, 35]. For example, inter-individual and/or sex differences occur in the processes underlying trigeminal nociceptive primary afferent excitability and in transmission and modulatory processes in trigeminal nociceptive pathways in the CNS; these and related features are discussed further in section 5.

### 3.2 Human studies

In the spinal system, human studies of acute or chronic pain have used a variety of *in vivo* and/or *in vitro* techniques to investigate pain-related behaviors (see section 2.2) as well as peripheral processes, neural pathways, CNS circuits, and mechanisms related to pain-sensorimotor interactions. These techniques have included clinical, electrophysiological, psychophysical, psychological, imaging, microdialysis, immunohistochemical, and genetic approaches, and have also been used in the analogous studies of acute and chronic orofacial pain outlined below [14, 15, 18, 20, 21, 38, 64, 65, 66]. The findings from these studies have shown that many CNS areas, including sensorimotor areas, play a role in the experience and modulation of pain, and, in general terms, they are comparable to the CNS areas outlined above in animal models of pain. Consistent with findings in animal models, many of these studies have provided evidence of peripheral and central sensitization, as well as neuroplasticity and glioplasticity occurring within these CNS areas, and some studies have shown alterations in sensorimotor behaviors including pain-related disruption of learning sensorimotor skills. Indeed, the plastic changes observed in patients with chronic pain are likely to have compromised the patient’s adaptive capacity and indicate a maladaptive plasticity that disturbs the normal functions of these areas and their related pathways. There is also evidence that CNS plastic changes induced by peripheral noxious stimuli may not always be readily reversible following alleviation of the peripheral afferent nociceptive input [2, 74]. Sex and inter-individual differences and age-related effects have also been noted in some of these influences and processes.

In the case of acute orofacial pain, use of techniques analogous to those used in the spinal system has revealed that tissue injury or inflammation induces the release of chemical mediators that enhance or reduce the excitability of peripheral nociceptors, and that many subcortical areas (*e.g.*, VBSNC, PAG, RVM) and cortical areas (*e.g.*, S1, S2, M1, insula, ACC, PFC) play a role in the multi-dimensional experience of pain including sensorimotor behaviors related to pain [1, 5, 6, 67, 75, 76]. Consistent with findings in the spinal system from studies in animal models and humans has been the documentation of neuroplastic and/or glioplastic changes induced in many of these CNS areas by acute orofacial noxious stimulation in humans. Since some of these changes may persist after alleviation of the peripheral afferent nociceptive input [2, 74], they may contribute to persistence of pain-related sensorimotor changes. Furthermore, learning of a novel orofacial sensorimotor task as well as the accompanying neuroplasticity in the face sensorimotor cortex may be disturbed in the presence of experimentally induced acute orofacial pain, which itself, as noted above, can induce plasticity [5, 21, 67, 77]. How these two sets of neuroplastic changes interact (*i.e.*, the plasticity associated with learning and the maladaptive plasticity that may be associated with acute pain) warrants further study because of its significance to understanding the processes whereby individuals can adapt or not to orofacial pain.

It is also important to note that, consistent with findings in animals, neuroplasticity and glioplasticity also occur in the human brain following other orofacial manipulations in addition to pain [5, 21, 77, 78]. Plastic changes within CNS circuits involved in nociceptive transmission, modulation or sensorimotor control may result from some common dental procedures and therapeutic approaches (*e.g.*, local anesthesia, tooth extraction, dental restorations, complete dentures, dental implant-supported prostheses) and may influence pain-sensorimotor interactions. Some of these environmental factors possibly act through epigenetic processes as outlined below in section 5.

In regards to chronic orofacial pain, application of techniques like those noted above in humans have been used in studies of pain states such as TMD, trigeminal neuralgia, BMS, and/or migraine headache [42, 43, 67, 76, 77, 79, 80, 81, 82, 83]. Some of these studies have documented that several chemical mediators may be released in peripheral tissues in chronic orofacial pain states and affect the excitability of nociceptors. Further, there are reports of activation, de-activation, glioplasticity, neuroplasticity and/or changes in white matter tracts or gray matter volume or thickness in one or more CNS areas involved in sensorimotor control, pain modulation, sensory-discriminative, motivational-affective, and cognitive-evaluative functions related to orofacial pain and sensorimotor behaviors (*e.g.*, VBSNC, thalamus, face sensorimotor cortex, ACC, dorsolateral PFC (dlPFC)) [43, 67, 76, 77, 79, 80, 82, 83]. Furthermore, some of the CNS changes correlate with several pain-related clinical measures (*e.g.*, pain intensity) which in chronic myofascial TMD patients can be reduced by high-definition transcranial direct current stimulation of face sensorimotor cortex that also increases pain-free mouth opening [84]. TMD patients may also show reductions in functional magnetic resonance imaging (fMRI) measures of adaptability to incoming afferent inputs in face sensorimotor cortex and the dlPFC [80] that may reflect a disruption of sensorimotor, pain-modulatory, and cognitive functions in TMD patients. Indeed, some of these CNS changes may be maladaptive in nature and may disrupt or compromise normal function and the ability of patients to adapt to the pain. For example, in some chronic orofacial pain states (*e.g.*, trigeminal neuralgia, BMS, TMD), significant structural and/or functional plastic changes noted in several CNS areas (*e.g.*, face M1, insula, dlPFC, ACC, PAG), white matter tracts, and their functional connectivity may disrupt the important roles of these areas and tracts in pain modulation and/or sensorimotor, sensory-discriminative, motivational-affective, and cognitive-evaluative functions [26, 76, 83]. Further evidence indicates that disturbances in the effects of the pain-modulatory pathways may play a critical role in the development and maintenance of chronic pain states. This comes from findings that enhanced temporal summation, reduced CPM (the human psychophysical parallel of DNIC in animals) and reduced blink reflex habituation may occur in some patients with chronic orofacial pain (*e.g.*, TMD, tension-type headache, persistent dentoalveolar pain, BMS, fibromyalgia) [26, 53, 70, 81].

In accord with the observations in animal models are findings in humans of variability between individual subjects in the changes observed in the CNS and in psychophysical or electrophysiological properties of subjects with chronic orofacial pain, some of which correlate with pain-related sensorimotor behaviors or clinical symptoms [8, 53, 80]. Age-related changes and sex differences in some of these features also have been demonstrated in humans as well as animal models; these include, for example, differences in some structural and functional features of sensorimotor and motivational-affective CNS areas and in electrophysiological or psychophysical parameters across one or more chronic orofacial pain states (*e.g.*, TMD, BMS, persistent idiopathic facial pain, migraine, cluster headache) [6, 15, 20, 31, 32, 67].

In summary, both animal models and human studies have documented the peripheral processes and trigeminal pathways and CNS processes underlying the multiple dimensions of orofacial pain, pain modulation, and sensorimotor and related behaviors. Differences between individuals, including in their age or sex, in some of these processes may contribute to inter-individual and sex differences between patients in orofacial pain features and associated sensorimotor behaviors. Pain-related neuroplastic and glioplastic changes in these processes are fundamentally involved in the expression of orofacial pain and associated sensorimotor behaviors and in adaptation or not to the pain. Indeed, many of the plastic changes in chronic orofacial pain states likely reflect a maladaptive plasticity that may disrupt or compromise pain-related sensorimotor behaviors. Such findings underpin major elements of the mechanistic framework of the TOPSMI by way of its articulation of the key role that CNS circuits and their neuroplasticity and glioplasticity play in adaptive and maladaptive processes and the interactions between orofacial pain and associated sensorimotor behaviors. There is evidence that pain-related sensorimotor behavior as well as related nociceptive circuits and processes are modified by significant modulatory influences from a variety of psychosocial factors, and this evidence is reviewed in the next section.

## 4. Psychosocial factors that influence pain-sensorimotor interactions

### 4.1 Animal models

Many studies of the spinal system in animals have revealed significant associations between measures of some psychosocial factors (*e.g.*, stress, anxiety, depression, changes in cognitive and environmental factors, pain-related fear) and sensorimotor behaviors associated with pain and nociceptive processes [14, 17, 85]. For example, stress can have complex influences on pain-related sensorimotor behaviors as well as nociceptive mechanisms. The duration and intensity of the stressor are particularly influential: short-duration, intense stress usually produces inhibitory effects on nociceptive circuits and their plasticity and related behaviors, while repeated or prolonged exposure to psychological or physical stress usually results in facilitatory effects. Other influential factors are the type of the stressor (*e.g.*, footshock or social conflict), the sex, age and strain of the animal, and environmental factors such as diet, sleep disruption, housing features for the animals, and even features of the experimenter. Some psychosocial factors (*e.g.*, chronic stress, adverse early life experiences) may also be associated with maladaptive plasticity in CNS areas contributing to social, cognitive-evaluative, emotional and sensorimotor functions, and may also influence properties of the musculoskeletal tissues used in sensorimotor behaviors [37, 86, 87, 88].

Psychosocial factors have also been shown to play a role in influencing nociceptive and sensorimotor circuits and related behaviors in acute orofacial pain models in animals. Stress in particular has been studied, although the mechanisms whereby stress may affect CNS circuits involved in sensorimotor behavior related to orofacial pain are not well understood. It has been shown that repeated forced swim stress conditioning or chronic restraint stress conditioning may induce orofacial allodynia or hyperalgesia that manifests as changes in sensorimotor behaviors (*e.g.*, head withdrawal responses to noxious orofacial stimuli) and associated EMG activity [89, 90, 91, 92, 93]. Repeated forced swim stress conditioning may also result in neuroplastic changes in responses of Vc/C1-2 neurons to intra-TMJ injection of ATP [92] or to masseter muscle noxious stimulation [94], and restraint stress may induce Vc glioplasticity in association with mechanical masseter allodynia and related sensorimotor behavioral changes [89, 91]. Different acute or chronic restraint stress protocols may however have different effects; for example, only a chronic restraint stress protocol results in hyperalgesia manifesting as significantly greater flinching and rubbing behaviors in response to TMJ injection of formalin [90]. Furthermore, these stressor paradigms may also result in increased anxiety (*e.g.*, assessed through the elevated plus maze test) or depression (*e.g.*, assessed through immobility time lengths in repeated forced swim stress conditioning), and associations have been noted between measures of depression or anxiety and sensorimotor behavioral measures of hyperalgesia [92, 94].

The effects of psychosocial factors on sensorimotor behaviors in chronic orofacial pain models have not been extensively reported. However, one study in a rodent neuropathic pain model (infraorbital nerve chronic constriction) has reported that chronic restraint stress results in sensorimotor behavioral changes indicative of allodynia and hyperalgesia [93]. Another series of studies using animal models of TMD comorbid with other conditions (*e.g.*, fibromyalgia or irritable bowel syndrome) reported that ovariectomized rats injected with estradiol exhibited long-lasting and widespread increases in mechanosensitivity (manifesting as reduced limb and head withdrawal thresholds to mechanical stimulation) induced by prolonged masseter inflammation together with repeated forced swim stress conditioning [25, 29]. The widespread increases in mechanosensitivity in these models may reflect a dysregulation of the balance between facilitation and inhibition in the descending pain-modulatory pathways, and likely result from plastic changes in these pathways. This is consistent with findings in the spinal system of facilitatory effects of repeated psychosocial stress on nociceptive circuits and plasticity that may result in altered sensorimotor behaviors [14, 17, 26, 67, 85]. However, in contrast to the spinal literature, there is very limited information from chronic as well as acute orofacial pain models as to the roles of other psychosocial influences (*e.g.*, animal age, sex and strain, and environmental factors such as diet, sleep disruption, and features of the housing room and the experimenter) on pain and pain-sensorimotor interactions, and this is an avenue requiring further research.

The possible role of age-related, inter-individual and sex differences in the influences that psychosocial factors may exert on sensorimotor behaviors associated with orofacial pain has also not been studied in detail in animal models. Although inter-individual differences were not specifically studied, the distribution of outcome measures about the mean in a group of animals subject to the same experimental protocol in some of the reports noted in section 2.1 in acute and chronic animal pain models may reflect variations between the individual animals in the group; these individual variations are considered further in section 5. In terms of possible sex differences, there is sensorimotor behavioral evidence in rats subjected to restraint stress of facial allodynia that persists for up to 3 weeks after the restraint stress protocol in female rats but not male rats [93]. Moreover, greater effects (*i.e.*, lower facial von Frey thresholds) in females than males of restraint stress were also found in rats having received a trigeminal nerve injury [93].

There is some information that psychosocial factors in animals can also influence orofacial musculoskeletal tissues themselves which could influence orofacial pain-sensorimotor interactions. For example, a chronic unpredictable mild stress protocol over several days has been shown to result in significant metabolic and vascular changes in the rat masseter and medial pterygoid muscles [37]. And it is well established that orofacial musculoskeletal tissues can be affected by environmental factors (*e.g.*, tissue injury, inflammation, diet, tooth loss, drugs, environmental pollutants, toxins, parafunctional activities) [25, 36, 88].

### 4.2 Human studies

In the spinal system, significant associations have been found between psychosocial factors (*e.g.*, stress, anxiety, depression, pain-related fear, economic factors, sociocultural and cognitive factors including pain catastrophizing, attention levels, expectations) and acute and chronic pain features such as pain-related sensorimotor behaviors, and/or neural activity in areas of the CNS involved in the cognitive-evaluative, motivational-affective, sensorimotor, and/or pain-modulatory aspects of pain [14, 15, 16, 18, 38, 65, 85, 95]. These associations may be bidirectional (*e.g.*, reciprocal influences between pain intensity and stress levels or cognitive processing), show age-related, inter-individual and sex differences, and vary with environmental changes or changes in other conditions. Some of the CNS areas may exhibit maladaptive plasticity in association with a range of psychosocial conditions (*e.g.*, post-traumatic stress disorder, stressful experiential factors such as stressful family environments and adverse early life experiences) which may influence subsequent nociceptive processing and sensorimotor behaviors related to pain. Furthermore, musculoskeletal tissues in addition to the sensorimotor systems controlling them may be influenced by psychosocial factors and may, thereby, alter pain-related sensorimotor behaviors [14, 37].

Psychosocial factors may also exert effects on acute orofacial pain and pain-sensorimotor interactions. Studies of experimental acute noxious stimulation (*e.g.*, hypertonic saline infusion evoking jaw muscle pain) show significant relationships between some psychosocial factors (*e.g.*, stress, anxiety, depression, pain catastrophizing) and orofacial pain-related sensorimotor behaviors (*e.g.*, changes in jaw muscle EMG activity or jaw movements during pain in comparison with pain-free control) and/or pain intensity scores [49, 96, 97]. It is also noteworthy that acute stress can be associated with changes in EMG activity recorded from facial, jaw and/or neck muscles in pain-free individuals [98, 99], suggesting that some psychosocial factors may influence pain-sensorimotor interactions not only via their effects on nociceptive processing (as suggested by the findings from the analogous animal studies mentioned above) but also via effects on the sensorimotor system *per se* [37, 86].

There have also been reports of significant associations between pain-related psychosocial factors (*i.e.*, pain catastrophizing, pain expectations) and structural and/or functional features of CNS areas and orofacial sensorimotor behaviors in individuals receiving experimental acute noxious stimulation [100]. For example, during jaw opening and closing in the presence of experimentally induced acute pain evoked in the masseter muscle (although not in the absence of pain), scores on the pain-catastrophizing scale significantly correlate with the amplitude of the signal intensity (on MRI) in CNS areas involved in orofacial sensorimotor behaviors (*e.g.*, the trigeminal motor nucleus, face M1, dlPFC, cerebellar cortex, posterior insula) [100]. In addition, in the presence of pain, the MRI signal intensity in the dlPFC exhibits a significant correlation with the variability in the amplitude and/or velocity of these repetitive open/close jaw movements [100].

In the case of chronic orofacial pain, it has been well-established that patients with a chronic orofacial pain state (*e.g.*, TMD, BMS, trigeminal neuralgia) exhibit significantly higher scores on measures of many psychosocial factors (*e.g.*, somatic awareness, affective distress, depression, anxiety, pain catastrophizing) in comparison with healthy controls, and some psychosocial factors may be risk factors for some chronic orofacial pain states [7, 53, 60]. Other studies have also documented significant relationships between some of these psychosocial measures and pain intensity scores, pain-related disability, and sensorimotor behaviors related to orofacial pain (*e.g.*, changes in face, jaw, or neck EMG activity or movements during pain) [59, 98, 99, 101]. For example, it has been reported that a group of pain-free individuals with a history of chronic trigeminal neuropathic pain exhibit significant negative correlations between pain-catastrophizing scores and jaw velocity and/or amplitude during chewing compared to a control group of pain-free individuals with no such history [59]. Some studies have also noted that the stress-related changes in jaw muscle EMG activity in some chronic orofacial pain states may be different from those in healthy pain-free controls, while other studies demonstrated no such differences [98, 99, 101]. The disparity in findings may be related to methodological differences between studies and/or to the contribution from other factors not considered in these studies, such as those noted in section 5.

Recent systematic reviews of chronic orofacial pain treatments have also provided findings consistent with the role of psychosocial factors in influencing pain-related sensorimotor activity [102, 103]. For example, psychological treatments (*e.g.*, relaxation treatment, cognitive behavioral therapy (CBT), cognitive coping skills training and/or hypnosis) in combination with standard treatments (*e.g.*, counselling, jaw exercises, occlusal appliances, pharmacological approaches) may produce greater increases in maximum jaw opening than standard treatment alone, and psychological treatments are as effective as standard treatments in reducing pain intensity in painful TMD [102]. While these systematic reviews indicate that psychosocial treatments may be valuable in contributing to reductions in pain intensity and improving jaw function in TMD patients, both reviews indicate that the experimental designs in some of the reviewed studies point to the need for further properly controlled studies to confirm these findings [102, 103]. In addition, further studies are needed to provide information on what psychosocial treatments are optimally suited to different TMD diagnoses and other orofacial pain conditions.

No fMRI studies are apparent of psychosocial associations in chronic orofacial pain individuals performing an orofacial sensorimotor task. However, significant associations have been reported between some psychosocial factors (*e.g.*, pain catastrophizing, pain expectations, pain self-efficacy) and structural and/or functional features of CNS areas (*e.g.*, gray matter volume, resting state activity, or connectivity between CNS areas) involved in the sensory-discriminative and motivational-affective dimensions of pain, pain modulation, and/or sensorimotor control in individuals with chronic orofacial pain such as TMD, migraine, and BMS [95]. Taken together, these CNS findings suggest that psychosocial factors may influence neuroplasticity in CNS areas in chronic orofacial pain and that some of these changes may reflect a maladaptive plasticity.

As in the animal models, there is very limited information as to the possible role of inter-individual differences in the influences of psychosocial factors on acute or chronic orofacial pain-sensorimotor interactions in humans, and it is not clear if age-related and sex differences occur. Nonetheless, given the variability both between and within studies as to the presence, magnitude and direction of any significant associations, the studies reviewed above do suggest inter-individual differences in the associations between acute or chronic orofacial pain-related sensorimotor behavior and psychosocial factors. While some of this variability may be attributed to methodological differences, inter-individual differences arising because of possible influences (*e.g.*, genetic and environmental factors) not addressed specifically in these studies are also likely to have affected the sensorimotor behaviors related to pain and are considered further in the next section. It is also important to note that the studies of psychosocial influences on orofacial pain and pain-sensorimotor interactions do not report causal relations but only associations which, when statistically significant, may demonstrate positive or negative correlations between, for example, a psychosocial measure and jaw motor activity.

Psychosocial factors appear also to affect orofacial musculoskeletal tissues (including the teeth) in humans as well as animals, and so could influence orofacial pain-related sensorimotor behaviors. There is evidence, for example, of masseter hypertrophy (as well as increased jaw muscle EMG activity) and tooth wear in individuals who carry out bruxing activities, some of which may relate to increased levels of anxiety or stress. In addition, it is well established that orofacial musculoskeletal tissues can be affected by environmental factors (*e.g.*, tissue injury, diet, drugs, environmental pollutants and toxins, parafunctional activities) [8, 62, 88, 104, 105].

In summary, the effects of psychosocial influences on orofacial pain and associated sensorimotor behaviors have not been extensively investigated, and further research is needed to aid clinical diagnosis and management. Nonetheless, the above outline of studies in animal pain models and humans does point to significant psychosocial influences on orofacial pain features, pain-related sensorimotor behaviors, nociceptive processes and plasticity. There is limited information on age-related and inter-individual and/or sex differences in the influences that psychosocial factors may have on acute and chronic sensorimotor behaviors related to orofacial pain in animal models and human studies. There is evidence in animals and humans for effects of psychosocial factors on orofacial musculoskeletal tissues; such effects might thereby influence pain-related effects on orofacial sensorimotor behavior. These findings collectively are consistent with major elements of the TOPSMI by providing evidence, albeit limited in some respects, for the important role that psychosocial factors play in pain and pain-sensorimotor interactions, in CNS plasticity and maladaptive plasticity, and in musculoskeletal structure and function. The next section reviews the evidence that genetic, epigenetic, and allied environmental factors also play a significant role in influencing sensorimotor behavior related to pain as well as nociceptive pathways and circuits in the CNS.

## 5. Orofacial genetic and epigenetic factors that influence pain-sensorimotor interactions

### 5.1 Animal models

Animal studies in the spinal system have contributed most of the findings relating to the roles that genetic, epigenetic, and allied environmental factors play in acute and chronic pain and pain-sensorimotor interactions, and in accounting for age-related, inter-individual, and sex differences in these interactions and underlying mechanisms [14, 17, 18, 38, 106]. While genetics refers to the study of genes and genetic variations (gene polymorphisms), epigenetics are reversible changes to the genome in the absence of changes to the nucleotide sequence; these reversible changes can be brought about by DNA methylation, histone acetylation, and noncoding RNA interference, although epigenetic patterns are modifiable by psychosocial factors (*e.g.*, psychological stress, lifestyle) and environmental factors (*e.g.*, injury, inflammation, environmental pollutants and toxins). Differences between strains and sexes have been well-described in nociceptive sensorimotor behaviors evoked by noxious stimuli, and such differences may reflect different single nucleotide polymorphisms (SNPs) and pain-related genes between sexes and strains. Epigenetic processes have also been demonstrated to regulate genes involved in nociceptive sensorimotor behaviors and in the plasticity and descending pain-modulatory pathways affecting the behaviors. Further, epigenetic processes have been implicated in the linkage between environmental factors (*e.g.*, injury, inflammation, environmental pollutants and toxins) and changes in gene expression, and a host of environmental factors can influence nociceptive sensorimotor behaviors. Genetic, epigenetic, and allied environmental factors can also influence the structural, functional and metabolic properties of skeletal muscles and bones in the spinal system, and could thereby influence sensorimotor behaviors related to pain.

By contrast with the spinal system, comparable data sets in animal models of the role of these factors in the orofacial nociceptive and sensorimotor systems are much more limited and more studies are warranted. Nonetheless, in the case of acute pain, there is evidence that genetic factors play a role in inter-individual and sex differences in CNS nociceptive processes and plasticity, and in acute orofacial pain-related sensorimotor behaviors, although there has been limited study of the factors influencing these differences. For example, as noted earlier (sections 2.1 and 3.1), differences between the sexes have been documented in nociceptive afferent and jaw muscle reflex activities following algesic chemical injection into TMJ or jaw muscle, as well as in the processes underlying CNS transmission and modulation of the trigeminal nociceptive afferent inputs and their pathways in the CNS; furthermore, some of these processes may be dependent on the level of sex hormones [6, 22, 31, 32, 33, 107]. Evidence for the role of epigenetic factors in sensorimotor behaviors in acute orofacial pain is suggested by findings that masseter muscle inflammation induces an acute (as well as persistent) decrease in DNA methylation in the trigeminal ganglion [108] that has been shown in other studies to be associated with significant reductions in head withdrawal thresholds to orofacial stimulation [109]. With regards to environmental factors, psychosocial factors play a role (see section 4) and several studies have shown that a variety of nutrients (*e.g.*, vitamins, minerals, omega-3 fatty acids, polysaccharides) may influence nociceptive processes within VBSNC and pain-related behavioral responses to noxious stimulation in animal models [88, 89].

With regard to chronic orofacial pain and sensorimotor behaviors, findings from a trigeminal neuropathic pain model in genetically different rodent strains subject to the same environmental conditions have revealed differences between strains in orofacial nociceptive sensorimotor behavior as well in VBSNC glioplasticity and neuroplasticity contributing to trigeminal central sensitization [6, 110, 111]. Genetic factors have also been implicated in the strain differences in the changes in volume in several CNS regions, which include those involved in sensorimotor and psychosocial functions in mice subjected to tooth extraction [6, 72]. Moreover, sex-dependent gene expression changes have been reported in the trigeminal ganglion in rats manifesting nociceptive sensorimotor behavior in a trigeminal neuropathic pain model [111]. These findings suggest that genetic factors are involved in the inter-individual variability and sex differences reported in orofacial nociceptive processes and sensorimotor behaviors. Moreover, there are emerging datasets that epigenetic factors also could be involved in the inter-individual variability and sex differences documented in orofacial nociceptive processes, plasticity and pain-related sensorimotor behaviors. For example, orofacial muscle inflammation resulting in significant long-term reductions in head withdrawal mechanical threshold is associated with changes in DNA methylation or upregulation of several nociceptive genes related to transient receptor potential subtypes vanilloid 1 (TRPV1) and ankyrin 1 (TRPA1), purinergic receptor subtype P2X3, brain-derived neurotrophic factor (BDNF), and piezo receptor subtype 2 (PIEZO2) in the trigeminal ganglion [108, 109]. Trigeminal neuropathic pain models also display epigenetic modifications (*e.g.*, involving histone deacetylases) in trigeminal ganglion neurons and/or central amygdala in association with changes in gene expression, CNS plasticity, and nociceptive sensorimotor behaviors [112, 113, 114]. Furthermore, reduced functioning of histone deacetylase inhibitors such as Sirtuin protein (SIRT1) in the central amygdala has been reported to be associated with the development of comorbid anxiety and depression and changes in orofacial sensorimotor behaviors in a trigeminal neuropathic pain model; the SIRT1 changes represent an epigenetic mechanism underlying individual pain vulnerabilities to emotional disorders [113].

The epigenetic studies reviewed above also provide examples of environmental factors (*e.g.*, masseter muscle inflammation, trigeminal nerve injury) resulting in epigenetic-related changes in gene expression that may influence orofacial pain-related sensorimotor behaviors. Another example of the close interplay of epigenetic and environmental factors is the evidence that adult male rats who as pups were separated from their dams exhibit orofacial mechanical allodynia manifesting as significantly lower head withdrawal thresholds to facial mechanical stimuli compared to adult male rats who as pups were left with their dams [115]. The authors reported that the allodynia may depend on increases in neonatal corticosterone signaling and the number of P2X3 receptor-immunoreactive trigeminal ganglion neurons [115], changes likely mediated at least in part via epigenetic mechanisms influencing plasticity in nociceptive pathways. Further research is needed to determine if other epigenetic and environmental factors documented in the spinal sensorimotor system (*e.g.*, environmental pollutants and toxins) also have roles in influencing chronic as well as acute orofacial pain-sensorimotor interactions in animal models.

It is also important to note that some environmental as well as epigenetic and genetic factors may influence orofacial musculoskeletal tissues in animals, and as a result could affect orofacial pain-sensorimotor interactions. For example, musculoskeletal changes have been well-documented in congenital disorders affecting the orofacial region [116, 117], and mouse genetic models for TMJ development and disorders are available [117]. Environmental factors such as the level of daily muscle use, diet, and unilateral extraction of the upper molars can influence muscle mass, muscle metabolic activity, and muscle fiber types in jaw muscles [36, 37, 118].

### 5.2 Human studies

Studies in the spinal system of humans have documented an important role of genetic, epigenetic, and allied environmental factors in pain experience and pain-sensorimotor interactions [14, 15, 18, 38]. For example, genetic factors (*e.g.*, SNPs) contribute to age-related, inter-individual and sex differences in several features of experimentally evoked acute pain, including sensorimotor behaviors, and to the female preponderance in many chronic pain states. A role for epigenetic factors is indicated by findings such as changes in microRNA expression or DNA methylation in individuals with fibromyalgia, neuropathic pain or irritable bowel syndrome. Moreover, epigenetic processes link genetic factors with a wide range of environmental factors including diet, toxins, prior pain experience, adverse childhood events, levels of physical activity, sociocultural factors, injury, and inflammation. Furthermore, some environmental factors may be involved in the age-related, inter-individual and sex differences in pain features and pain-related sensorimotor behaviors through epigenetic processes influencing CNS plasticity. Gene-environment interactions occurring where two different genotypes respond in different ways to environmental variations appear also to be involved in some states of chronic pain. In addition, genetic, epigenetic, and allied environmental factors may alter sensorimotor behaviors related to pain through their influences on musculoskeletal structure and function in the spinal system.

Studies of acute pain in the orofacial nociceptive and sensorimotor systems of humans also point to an important role of genetic, epigenetic, and allied environmental factors in the inter-individual variability and age-related and sex differences in orofacial pain features, including pain-sensorimotor behavior [6, 15, 46, 58, 60, 119, 120, 121, 122, 123, 124]. For example, women can exhibit significantly greater pain intensity scores than men for acute pain experimentally induced by glutamate injection into the masseter muscle, and while pre-pain baseline jaw-closing reflex EMG responses have been reported to be larger in women than men, glutamate injection significantly facilitates these EMG responses in men but not women [46, 58]. Since facilitated jaw-closing reflex responses may serve to reduce jaw mobility and thus protect against further injury, this latter finding suggests that women might have less protection against exacerbation of an existing jaw muscle injury than men [46]. However, another study of experimental masseter muscle pain reported no significant associations between sex and pain-related changes in jaw muscle EMG activity [48]; the small sample size in this study may have contributed to this negative finding. The specific involvement of genetic factors in inter-individual as well as sex differences in effects of experimental acute noxious stimulation is supported by evidence for these differences in some genetic associations with orofacial pain intensity. For example, SNPs of the catechol-O-methyltransferase (*COMT*) gene may result in different levels of activity of the enzyme (catechol-O-methyltransferase) that metabolizes catecholamines [122]. The SNPs of the *COMT* gene have an association with pain intensity scores recorded during experimental acute noxious stimulation (*e.g.*, heavy pressure to facial skin overlying jaw muscles, capsaicin application to facial skin), and they may explain some of the inter-individual variability in the sensitivity to experimental acute noxious stimulation [124]. Associations also exist between SNPs of genes related to opioid, catecholaminergic, inflammatory, and/or dopaminergic pathways and sensitivity to acute noxious orofacial thermal and pressure stimuli in healthy pain-free individuals [123], and between SNPs of the *COMT* gene and toothache-related pain intensity in children [125]. There are also associations between many SNPs and risk of TMD onset (findings from the Orofacial Pain: Prospective Evaluation and Risk Assessment (OPPERA) project) [60, 122]. These differences in SNPs between individuals may contribute to differences in acute pain features including pain-related sensorimotor behaviors by being associated with inter-individual differences in plasticity along nociceptive and pain-modulatory pathways. Furthermore, the SNPs encoding for low catechol-O-methyltransferase activity have been shown to be associated with increased acute pain intensity in response to capsaicin application to the facial skin in females, but not males [124].

While there is only limited information of epigenetic processes and environmental factors in acute orofacial pain and related sensorimotor behaviors in humans [6, 120, 122, 126, 127], studies to date point to epigenetic processes, and environmental factors analogous to those noted above in the spinal literature are also involved in acute orofacial pain and related sensorimotor behaviors. For example, evidence for epigenetic influences comes from findings that dynamic regulation of DNA methylation patterns in the genome located near genes regulating inflammatory and neuronal responses may occur during development or resolution of recent onset acute TMD [126]. Furthermore, environmental factors may also have an effect on acute orofacial pain; for example, anxiety reflects an environmental stressor often associated with dental procedures that may influence dental pain [127].

In the case of chronic orofacial nociceptive and associated sensorimotor behaviors in humans, an important role for genetic factors is supported by observations that several chronic orofacial pain states (*e.g.*, some neuropathic pain states, migraine headache, TMD) have a female predominance, or may exhibit inter-individual or age or sex-related differences in CNS structure and function or in pain features including pain-related sensorimotor behaviors; other factors (*e.g.*, environmental) as well as genetic factors may contribute to these differences [15, 38, 53, 60, 122, 128, 129]. In addition, TMD and some orofacial neuropathic pain states (*e.g.*, BMS, trigeminal neuralgia) may be associated with genetic variations in ascending nociceptive and/or descending pain-modulatory pathways. For example, the OPPERA project has identified several SNPs of genes relevant to nociceptive, pain-modulatory and affective neural pathways that appear to be linked to TMD and associated symptoms [60, 122]. Different SNPs may be associated with differences between individuals in plasticity along nociceptive pathways and thereby may play a role in the variability between individuals in pain features and pain-sensorimotor interactions. Furthermore, some gene variants (*e.g.*, variants of the *COMT* gene) exhibit associations with the susceptibility to the development of TMD, sensitivity to noxious stimulation of the masseter muscle, and effects of therapeutic interventions on pain and mouth opening in TMD patients [119, 122, 130, 131]. For example, a recent study of 60 patients with pain-related TMD has shown that certain SNP variants of the gene (opioid receptor mu 1 (*OPRM1*)) that encodes the opioid receptor mu 1 protein or the *COMT* gene are associated with a poorer treatment response to standard TMD treatments (*e.g.*, information and education, home physical therapy, occlusal splint) in respect to pain-free mouth opening, pain intensity and anxiety [130].

Investigations of epigenetic and allied environmental factors on chronic orofacial pain features including sensorimotor behaviors in humans have revealed that some chronic orofacial pain states (*e.g.*, TMD, persistent idiopathic facial pain, persistent dentoalveolar pain) exhibit significant gene-environment interactions. These interactions involve epigenetic factors that may be involved in the inter-individual variability and sex differences in pain features and pain-sensorimotor interactions that have been documented in these chronic orofacial pain states [6, 53, 60, 119, 121, 122, 132]. It is also noteworthy that the influence of SNPs or gene variants (*e.g.*, *COMT*) on the development of painful TMD or on pain features can be modified by psychosocial factors (*e.g.*, stress, depression), thus reflecting gene-environment interactions that possibly reflect psychosocial factors influencing plasticity in nociceptive pathways involving these genes. Dynamic regulation of genes in response to environmental exposures (*e.g.*, injury, psychological stress) may be an important mechanism contributing to the risk of developing chronic painful TMD [126]. Other environmental factors may represent risk factors for TMD or may correlate with pain intensity ratings or sensorimotor behaviors in TMD patients; these environmental factors include history of trauma to the lower jaw, parafunctional activities, presence of other pain states [60, 133], socioeconomic status [134], psychoactive substance abuse [135], and dietary supplements (*e.g.*, vitamins, minerals, glucosamine, polysaccharides) [88].

As in the animal models, genetic, epigenetic, and allied environmental factors are also involved in humans in influencing orofacial musculoskeletal tissues and may therefore influence pain-sensorimotor interactions. For example, genes play an important role in determining orofacial skeletal form [116], and sex or inter-individual differences have been reported for masticatory muscle attachment morphology, muscle tendon architecture and muscle fiber type distribution [136]. While some of these differences likely reflect genetic variations, epigenetic and allied environmental factors are also likely involved, such as the levels of jaw motor activity associated with, for example, tissue injury, dentitional state, orofacial skeletal form, age, and the rheological properties of ingested food [56].

In summary, the findings outlined above from animal and human studies support the view that genetic, epigenetic, and allied environmental factors are significant in the experience of orofacial pain and related behaviors, including pain-sensorimotor interactions. These factors are also important in accounting for age-related, inter-individual and sex differences in these interactions. There is also evidence for several of these factors influencing nociceptive, descending pain-modulatory, and sensorimotor CNS areas, and musculoskeletal tissues themselves. These findings are consistent with major elements of the TOPSMI that emphasize the important contribution of genetic, epigenetic, and allied environmental factors to pain and pain-sensorimotor interactions. The next section synthesizes the various findings outlined in sections 2–5 and considers them for their fit within the framework of TOPSMI. Also considered are the clinical relevance of TOPSMI to orofacial pain-sensorimotor interactions as well as gaps in knowledge requiring future research.

## 6. Synthesis, and clinical implications and future research directions

Acute and chronic orofacial pain states are commonly associated with a wide range of changes in sensorimotor behavior, some of which are stereotyped (*e.g.*, reflexes) while others are more complex and can show considerable inter-individual and sex differences. Our understanding, diagnosis and management of pain-sensorimotor interactions have been dominated for almost a century by theories lacking a full picture of these interactions, especially the many processes and factors involved in the interactions. The present review of findings from animal and human studies indicates that the recently conceptualized TOPSMI provides a more encompassing view of orofacial pain-sensorimotor interactions and takes account of the many factors and processes shown to influence them. These include the important role played by glioplasticity as well as neuroplasticity in affecting the CNS circuits underlying orofacial pain processing and modulation and related sensorimotor behaviors. The findings also underpin TOPSMI’s emphasis on the importance of psychosocial, genetic, epigenetic, and allied environmental factors affecting the glioplasticity and neuroplasticity of CNS circuits, as well as the musculoskeletal tissues involved in the sensorimotor behaviors. Several of these factors are also important in the variability between individuals and age or sex-related differences in the expression of orofacial pain and related sensorimotor behaviors and to the ability of individuals to adapt (or not) their behaviors to the pain being experienced.

TOPSMI was formulated in accordance with the biopsychosocial model of pain, and Fig. 3 provides a conceptual schema illustrating that the processes and factors underlying orofacial pain-related changes in sensorimotor behavior are influenced by the integral features of the biopsychosocial model of pain, namely its biological, psychological, and sociocultural components. For example, in an individual with a “favorable” mix and weighting of psychosocial, genetic, epigenetic, and allied environmental factors, the CNS circuits have intrinsically the capacity for an adaptive plasticity state providing that individual with an array of pain-free adaptive options for appropriate activation patterns of motor units. In contrast, an individual with a particular mix and weighting of adverse or “unfavorable” biopsychosocial factors (*e.g.*, high psychosocial distress; poor genetic and epigenetic profile, poor anatomical form, negative sociocultural factors) may have sensorimotor, nociceptive and modulatory neural circuits that may be “set” to a maladaptive plasticity state that, in the presence of pain, leads to maladaptive motoneuron outputs. These perspectives are also consistent with the proposal of TOPSMI which highlights that various psychosocial, genetic, epigenetic, and allied environmental factors not only may influence CNS processes related to orofacial pain and related sensorimotor behaviors but also orofacial musculoskeletal tissues themselves and thereby influence pain-sensorimotor interactions.

**Fig. 3.** Conceptual framework linking the Theory of Pain-Sensorimotor Interactions (TOPSMI) with the biological, psychological, and sociocultural components of the biopsychosocial model of pain. The examples of biological, psychological, and sociocultural components contributing to the biopsychosocial model of pain are shown in relation to orofacial pain-related CNS activity and plasticity and influences on adaptive or maladaptive sensorimotor behavior. These components, and the interactions among the components, can influence orofacial pain sensorimotor behavior, resulting in a mosaic of behavioral features that may differ from one individual to another. CNS: central nervous system.

The TOPSMI may also have other implications for individuals with pain, by informing diagnosis and management. The theory suggests that individuals with different clinical orofacial pain states (*e.g.*, BMS, TMD, persistent dentoalveolar pain) will have different mixes and weightings of various biological, psychological, and sociocultural factors (as well as interactions among factors) that will not only influence orofacial pain but also contribute to the diversity of orofacial pain-related sensorimotor behaviors and comorbidities (*e.g.*, sleep disruption, depression, movement limitations) between clinical orofacial pain states. If these sensorimotor behaviors can be categorized, possibly through AI assistance, and linked with specific orofacial pain states and with specific mixes and weightings of factors, then this may assist in diagnosis and management by providing likely options for differential diagnoses and management. Such perspectives stemming from TOPSMI draw attention to the need for orofacial pain patients, especially those experiencing chronic pain, to have access to comprehensive diagnostic approaches that extend their focus beyond peripheral or oral (environmental) factors and influences such as injury or inflammation. These approaches need to take into account the contribution to the patient’s pain and any associated maladaptive sensorimotor behavior from CNS processes and plasticity and the influences of biological, psychological, and sociocultural factors. The perspectives also reinforce the importance of broadening the focus of management from just targeting the peripheral tissues (*e.g.*, teeth, TMJs, jaw muscles) to include other approaches harnessing CNS mechanisms including plasticity so as to shift CNS circuits from a maladaptive state disrupting normal CNS functions to circuits that can adapt to pain without the development of further pain and sensorimotor disruption. These approaches could include, for example, one or more of physical therapy, counselling, analgesic, anti-depressive or sleep medications, psychotherapy, or hypnosis, in association with standard dental treatments [7, 67, 137], but their selection would depend on the particular clinical features of the patient and their pain state. Indeed, in accord with recent studies advocating for customized management for chronic pain [7, 14, 15, 16, 53, 60, 122], these perspectives also point to the need for customized chronic pain management strategies by acknowledging an individual’s particular mix and weighting of psychosocial, genetic, epigenetic, and allied environmental factors and processes, rather than treating all patients experiencing the same particular chronic orofacial pain state with exactly the same generic management strategy that doesn’t acknowledge the individuality of the pain experience.

Fig. 4 outlines TOPSMI’s mechanistic framework from these various perspectives. The framework facilitates the understanding, for example, of how two individuals with a comparable pain-producing event may adopt different sensorimotor behaviors associated with different clinical outcomes: one individual able to minimize or even adapt to the pain, and a second individual experiencing no pain minimization or adaptation. The existing mix and weighting of psychological, sociocultural, genetic, epigenetic, and allied environmental factors influencing the first individual (as depicted for individual A in Fig. 4) have “set” the CNS circuits to an adaptive plasticity state providing a broad range of adaptive options available for appropriate motor unit activation patterns and adaptive sensorimotor behavior which are associated with a state of homeostasis and pain minimization or adaptation. However, an existing adverse mix and weighting of factors in the second individual (as depicted for individual B in Fig. 4) “set” the CNS circuits to a state favoring a maladaptive plasticity state and resulting in maladaptive sensorimotor behavior associated with no clinical improvement or even a worsening of the pain. For effective clinical management of this second individual, the clinician would need to consider the range of psychosocial as well as biological factors contributing to this individual’s condition. The TOPSMI also argues that management approaches only directed towards a single factor (*e.g.*, a dental occlusal interference) will not address the underlying mix and weighting of several factors that are likely contributing to either a maladaptive plasticity state or a plasticity state with few adaptive options available. Treatment should be directed to the full range of factors identified in a particular individual.

**Fig. 4.** Mechanistic framework based on the Theory of Pain-Sensorimotor Interactions (TOPSMI) and illustrating individual differences in the interactions between sensorimotor behaviors and pain. Features of TOPSMI are illustrated for their influences on clinical outcomes in two individuals (A on the left, B on the right) with a comparable pain-producing event such as ongoing jaw muscle pain following excessive jaw opening during a dental extraction procedure or recent-onset pain related to a traumatic dental injury. A “favorable” mix and weighting of biopsychosocial factors (*e.g.*, low psychosocial distress, good anatomical form, social and cultural factors, genetic and epigenetic profile) influence adaptive CNS circuits producing the pain-related sensorimotor behavior of individual A. The interaction of these factors (vertical bidirectional arrow in green) with the adaptive neuroplasticity and glioplasticity in nociceptive, modulatory and sensorimotor circuits elicits (oblique arrow in green) adaptive motoneuron outputs and activation patterns of motor units (upper vertical arrow in green, “b”) that may also influence or be influenced by features of the musculoskeletal tissues involved in the resulting adaptive sensorimotor behavior. These patterns involve changes in firing rates and recruitments of those motor units providing for the adaptive sensorimotor behavior at low metabolic cost and a level of biomechanical advantage so as to ensure an acceptable degree of homeostasis and allowing for reduction of or adaptation to any pain being experienced (lower vertical arrow in green, “b”, left side of lowest box). Other patterns of activation of motor units (*e.g.*, “a” and “c” and associated vertical dotted arrows in green) are not used by individual A since these options represent a less optimal blend of metabolic cost and biomechanical advantage in this individual for ensuring a degree of homeostasis and pain minimization comparable to that available with option “b”. Nonetheless, another individual who has a different mix and weighting of adaptive processes and favorable features could adopt one of the other options in order to have a comparable degree of homeostasis and pain minimization or adaptation. In contrast, individual B has an “unfavorable” adverse mix and weighting of biopsychosocial factors (*e.g.*, high psychosocial distress, poor anatomical form, social and cultural factors, genetic and epigenetic profile) associated with (vertical bidirectional arrow in red) maladaptive neuroplasticity and glioplasticity that lead (oblique arrow in red) to motoneuron outputs that are maladaptive and produce (vertical arrows in red, “e”) maladaptive patterns of activation of motor units that result in maladaptive sensorimotor behavior associated with no improvement in pain or even the development of new pain or worsening of pain (right side of lowest box). Another mix and weighting of unfavorable biopsychosocial factors may result in other possible maladaptive activation patterns of motor units and motoneuron outputs and other effects on pain (*e.g.*, “d” and “f” and associated vertical dotted arrows in red). The figure also shows that, subject to the weighting and mix of factors that an individual might be currently experiencing, shifts from unfavorable to favorable and from maladaptive to adaptive and *vice versa* (horizontal bidirectional arrows in black) can occur in an individual. Depiction based on elements of Fig. 3 in Murray and Sessle 2024 [14].

While we consider that TOPSMI has addressed many of the shortcomings of previous theories of pain-sensorimotor interactions, TOPSMI itself has some limitations in the case of orofacial pain-sensorimotor interactions. These largely stem from the limited understanding, at present, of orofacial nociceptive mechanisms and associated glioplasticity and neuroplasticity involved in orofacial pain-related sensorimotor behavior and the modulatory influences of psychological, sociocultural, genetic and epigenetic, and allied environmental factors. Another possible limitation of the TOPSMI is that while individual factors of the theory can be tested, so many factors appear to be involved in pain-sensorimotor interactions that the theory may be difficult to be formally tested as a whole, although this does not preclude the testing of individual factors in these interactions. Furthermore, because of the novelty of the TOPSMI (it was only published in 2024), there have not yet been any reported experimental studies specifically testing the applicability (or not) of TOPSMI to orofacial pain mechanisms and orofacial pain-sensorimotor interactions. There is thus the need in animal models and in humans for investigations of the peripheral and central nociceptive processes underlying orofacial pain, including neuroplastic and glioplastic processes, and the mechanisms by which various psychological, sociocultural, genetic, epigenetic, and allied environmental factors influence adaptative and possibly maladaptive pain-sensorimotor interactions as well as musculoskeletal tissues themselves. Such investigations would thus help in clarifying the applicability of TOPSMI to these mechanisms and factors and orofacial pain-sensorimotor interactions.

Further studies of these various mechanisms and factors should include behavioral as well as CNS, genome, and AI-based investigations to address their effects and the specific factors and processes underlying age, inter-individual and sex-related differences and the contributions of these differences to the susceptibility, development and expression of chronic orofacial pain and pain-related adaptive and maladaptive sensorimotor changes. Some of these study designs should include longitudinal neuroimaging trials, biopsychosocial stratification, or gene-environment interaction models in order to produce findings that facilitate advancements of knowledge in the field as well as operationalize the TOPSMI framework. Further research is also needed to develop improved methods of jaw, face, and tongue EMG recording, motion tracking, and CNS imaging in clinical and laboratory research settings to provide more comprehensive datasets from patients and controls, and to characterize the diversity of pain-related sensorimotor features in different orofacial pain states, and to assist in diagnostic stratification of patients. Studies are also needed to facilitate the synthesis of disparate datasets to discover novel mechanistic classification systems for improved diagnosis and management programs that shift the plasticity of orofacial sensorimotor networks from a maladaptive state to an adaptive state, and thereby restore normal sensorimotor behavior and alleviate pain.
